# Public attitudes to emergency care treatment plans: a population survey of Great Britain

**DOI:** 10.1136/bmjopen-2023-080162

**Published:** 2024-09-23

**Authors:** Martin Underwood, Angela Noufaily, Chris Bain, Jenny Harlock, Frances Griffiths, Caroline Huxley, Gavin Perkins, Sophie Rees, Anne-Marie Slowther

**Affiliations:** 1 Warwick Medical School, Coventry, UK; 2 University Hospitals Coventry and Warwickshire NHS Trust, Coventry, UK; 3 HealthWatch Coventry, Coventry, UK; 4 Bristol Trials Centre, University of Bristol, Bristol, UK

**Keywords:** medical ethics, surveys and questionnaires, patient-centered care

## Abstract

**Abstract:**

**Objectives:**

To measure community attitudes to emergency care and treatment plans (ECTPs).

**Design:**

Population survey.

**Setting:**

Great Britain.

**Participants:**

As part of the British Social Attitudes Survey, sent to randomly selected addresses in Great Britain, 1135 adults completed a module on ECTPs. The sample was nationally representative in terms of age and location, 619 (55%) were female and 1005 (89%) were of white origin.

**Outcome measures:**

People’s attitudes having an ECTP for themselves now, and in the future; how comfortable they might be having a discussion about an ECTP and how they thought such a plan might impact on their future care.

**Results:**

Predominantly, respondents were in favour of people being able to have an ECTP, with 908/1135 (80%) being at least somewhat in favour. People in good health were less likely than those with activity-limiting chronic disease to want a plan at present (52% vs 64%, OR 1.78 (95% CI 1.30 to 2.45) p<0.001). Developing a long-term condition or becoming disabled would lead 42% (467/1112) and 43% (481/1112) of individuals, respectively, to want an ECTP. More, 634/1112 (57%) would want an ECTP if they developed a life-threatening condition. Predominantly, 938/1135 (83%) respondents agreed that an ECTP would help avoid their family needing to make difficult decisions on their behalf, and 939/1135 (83%) that it would ensure doctors and nurses knew their wishes. Nevertheless, a small majority—628/1135 (55%)—agreed that there was a serious risk of the plan being out of date when needed. A substantial minority—330/1135 (29%)—agreed that an ECTP might result in them not receiving life-saving treatment.

**Conclusions:**

There is general support for the use of ECTPs by people of all ages. Nevertheless, many respondents felt these might be out of date when needed and prevent people receiving life-saving treatment.

**STRENGTHS AND LIMITATIONS OF THIS STUDY**

- We recruited a nationally representative sample as part of the British Social Attitudes Survey.
- There was patient and public input into developing and refining survey questions.
- Some of those for whom emergency care and treatment plans may be most relevant, such as frail older people and those with cognitive impairment, might be less likely to respond to a ‘push’ survey of this nature.
- Minority ethnic groups are slightly under-represented among the respondents.

## Background

 Anticipating future clinical emergencies or sudden deteriorations in a person’s clinical condition and making recommendations for treatment decisions should this occur is challenging. However, absence of planning for future emergency treatments can result in individuals receiving treatment that is ineffective or harmful. The use of ‘Do Not Attempt Cardiopulmonary Resuscitation’ (DNACPR) recommendations for people with a life-threatening condition is well established. These are, however, unidimensional, failing to provide meaningful clinical information, or any information on the individual's wider treatment preferences. They also do not provide information on other treatment options that might help emergency or urgent clinical decision-making.^1^,^3^

Advance care planning addresses this concern by establishing, in a formal and holistic manner, recommendations about an individual’s future care to take effect in the event they lose capacity to make their own decisions.^4^ They can include preferences on wider aspects of future care, such as preferred place of death. Advance care planning is recommended as part of both the National Institute for Health and Care Excellence guidance and the UK national Gold Standards Framework for end-of-life care.^4^,^6^ The focus of advance care planning tends to be on end-of-life care. In contrast, emergency care treatment plans (ECTPs), or treatment escalation plans, focus on making recommendations to support medical decision-making in emergency or acute situation, or in the event of clinical deterioration from which recovery is possible, even if unlikely.^7^ Like advance care plans, they reflect discussion between the person, or their family if the person lacks capacity, and a clinician about the person’s preferences and the potential future treatment options. The resulting recommendations can encompass a range of treatments including cardiopulmonary resuscitation. The recommendations are intended to guide future treating clinicians when making treatment decisions in an emergency, although the responsibility for decision-making remains with the treating clinician.^8^

In 2019, 59% of NHS Acute Trusts were using some form of ECTP in addition to or instead of DNACPR forms.^9^ This transition is also occurring in primary care. In a survey (n=841) of general practitioners’ (GPs) attitudes towards the use of ECTPs, we found that they were very supportive of their use and that GPs considered that they provided benefits for patients. Around 80% of respondents were comfortable having ECTP discussions with patients or their families. While these plans were reported to be primarily completed by GPs, many respondents supported expanding the pool of healthcare and social care professionals who could complete ECTPs^10^. The most used model of ECTP in the UK is the Recommended Summary Plan for Emergency Care and Treatment (ReSPECT), introduced in 2016. Several studies have explored the experiences of, and attitudes to ReSPECT from the perspective of health and social care professionals, patients and their families.^11^,^16^ However, to our knowledge, there have been no large studies examining the views of the UK public on ECTPs in general, or ReSPECT in particular. During the development of ReSPECT, a national consultation exercise was conducted that included responses from 97 members of the public.^17^

While there is a survey of attitudes towards dying as part of the 2013 British Social Attitudes (BSA) survey, there is little quantitative research on the general public’s attitudes to ECTPs.^18^ A 2020 YouGov survey commissioned by ‘Compassion in Dying’ found that only a minority of people understood reasons behind DNACPR recommendations, or the treatment options available if a doctor had made such a recommendation.^19^ A 2021 mixed methods study of public attitudes towards advance care planning in Northern Ireland identified there was limited awareness and lack of knowledge about the process along with some misperceptions of its intent.^20^ We report here on a survey of residents in England, Scotland, and Wales (Great Britain) regarding emergency care treatment planning. The survey is part of a larger mixed methods project evaluating the use of ECTPs and ReSPECT in primary and community care.^21^ As part of the wider study, we ran focus groups with members of the public to seek their views. Overall, they were supportive of ECTPs as a means of normalising end-of-life discussions, emphasised the importance of putting the patient at the centre of the discussion, but expressed some doubt as to whether they would be acted on in an emergency.^21^ In the survey reported here, our specific objectives were to find out about: (1) people’s attitudes to wanting an ECTP for themselves now, and in the future; (2) how comfortable they might be having a discussion about an ECTP and (3) how they thought such a plan might impact on their future care.

## Methods

To ensure a high-quality nationally representative population sample, we commissioned the National Centre for Social Research to include our questions in the annual BSA survey.^22^ The BSA is the UK’s longest-running survey of public opinion; its first report was published in 1983. It is viewed as the authoritative barometer of public attitudes by government, academia and the media. It provides a high-quality nationally representative attitudinal survey with a stratified sample based on postcode and includes face-to-face data collection using computer-assisted personal interviewing and self-completion. To develop our questions, first study team members (A-MS, FG, SR, JH) developed a proposed set of questions drawing on data from focus groups we ran as part of our linked qualitative study. Following feedback from our lay advisory group these were refined. We then met with the BSA survey team to iteratively develop and arrive at the final draft set of questions. We originally planned to use 11-point numerical rating scales; however, the BSA team advised that in their experience 5-point Likert scales were more likely to be satisfactorily completed, and the findings would be easier to interpret.

A sample of these questions were then evaluated by the BSA team in 10 cognitive interviews and the full set of questions piloted in telephone interviews with 56 people, all recruited from the National Centre for Social Research’s panel database. Results of the cognitive interviews and pilot were discussed with the study team and minor modifications made to arrive at the final question wording. The final module included 15 questions of varying design (online supplemental appendix 1).

Data collection was carried out in accordance with the National Centre for Social Research’s protocol for delivering the BSA survey which is as follows. Invitations are sent to a stratified sample of households in the UK identified from the Royal Mail’s Postcode Address File with up to two adults from each address able to participate. There is an option of online or telephone interview completion. Two online access codes are provided to each household. For those not wanting to do the survey online, there is an option to call a freephone number for telephone completion with a specialist telephone interviewer. Up to three postal interventions are sent with a conditional £10 incentive to participate.^23^ For online completion, consent was implied by survey completion and for telephone interviews verbal consent was obtained from the interviewer prior to participation.

The information we sought included data on people’s attitudes to wanting an ECTP for themselves now, and in the future how comfortable they might be discussing an ECTP, and how they thought such a plan might impact on their future care. We drew basic demographic details from the core module of the BSA survey. The target sample size for our module was 1000 participants.

In addition to descriptive statistics for each question, we present logistic regression analyses investigating the covariates associated with three dichotomised dependent variables: people being in favour (strongly/somewhat) or not of anyone being able to have an ECTP; whether they would like (definitely/probably) or not an ECTP at present and (for those who did not already have an ECTP) how comfortable (very/fairly) or not they would feel about making such a plan for themselves. For each analysis, the independent variables were age, gender, ethnicity, educational level, having an illness lasting >12 months, caring for someone ill or with a disability and whether they are close to someone with a condition that could shorten their life. These were chosen after discussion within the study team to identify ‘a priori’ the most important possible independent variables. In each case, a fully adjusted model is presented.

### Patient and public involvement

Our lay advisory group contributed to the design of the overall project, commented on all patient and public facing materials, commented on initial study findings and participated in the overall study stakeholder meeting. Specifically for the public survey, they provided feedback and suggestions on draft questions during development. Author CB was a co-applicant on the original proposal.

## Results

The survey ran from 9 September 2022 to 30 October 2022. Of the 6699 respondents to the BSA survey, 1135 completed our module. Overall, they were broadley representative of Great Britain (GB) as a whole, although minority ethnic groups were under-represented (10.0% vs 13.5% of GB adults) (online supplemental appendix 2, table S1). Seventeen respondents (1.5%) currently had an ECTP in place. Nine of these were very comfortable (53%) and five (29%) were comfortable with making their plan. However, one was fairly uncomfortable and one was very uncomfortable with making their plan. The most common times people had ECTP forms completed were after diagnosis of a life-threatening illness (7/17) and after being told they had a long-term condition (6/17) (online supplemental appendix 2, table S2). Eight had been completed for people aged under 45 years and nine for people aged 45 years or over. Most plans were completed by the respondent’s GP (6/17) or another doctor who knew them well (4/17, 24%) (online supplemental appendix 2, table S3). A minority were completed by nurses at the GP surgery (2/17) (online supplemental appendix 2, table S3).

Predominantly, respondents were in favour of people being able to have an ECTP if they so wished, 908/1135 (80%) were at least somewhat in favour (table 1). In our regression analysis, males were less likely to be in favour than females 77% vs 82% (OR 1.45, 95% CI 1.06 to 1.97, p=0.020). When compared with those with no qualifications, people with degrees were more likely to be in favour; 75% vs 84% (OR 2.68, 95% CI 1.42 to 5.07, p=0.002) (table 2). Overall ethnicity did not affect people’s views. However, people of Asian ethnicity might be less likely than white people to be in favour of everyone being able to have an ECTP; 67% vs 82% (OR 0.45, 95% CI 0.25 to 0.84, p=0.012) (table 2).

**Table 1:** Are you in favour or against anyone being able to have an emergency care and treatment plan if they wish? (N=1135) n (%)

Strongly in favour	513 (45%)
Somewhat in favour	395 (35%)
Neither in favour nor against	189 (17%)
Somewhat against	20 (2%)
Strongly against	11 (1%)
Do not know/refused	7 (1%)

**Table 2:** Are you in favour or against anyone being able to have an emergency care and treatment plan if they wish?* (N=1135)

		In favour		P value	OR (95% CI)
Gender					
	Male	380/493	77%		1
	Female	510/619	82%	0.020	1.45 (1.06 to 1.97)
	Other	7/8	88%	0.382	2.62 (0.3 to 22.69)
Age (years)					
	18–24	51/66	77%	–	1
	25–34	137/173	79%	0.922	1.04 (0.51 to, 2.11)
	35–44	138/172	80%	0.748	1.12 (0.55 to 2.30)
	45–54	160/205	78%	0.955	1.02 (0.51 to 2.04)
	55–59	85/99	86%	0.140	1.89 (0.81 to, 4.40)
	60–64	86/107	80%	0.598	1.24 (0.56 to 2.73)
	65–69	82/107	77%	0.992	1.00 (0.47 to 2.17)
	70+	168/205	82%	0.295	1.48 (0.71 to 3.07)
Ethnicity					
	White	820/1005	82%	–	1
	Black	10/14	71%	0.285	0.52 (0.15 to, 1.73)
	Mixed	30/44	68%	0.115	0.57 (0.29 to 1.15)
	Asian	37/55	67%	0.012	0.45 (0.25 to 0.84)
Educational level					
	No qualifications	43/61	70%	–	1
	Qualifications less than A level	138/183	75%	0.358	1.37 (0.70 to 2.66)
	General Certificate of Education A-levels/Scottish Certificate of Education Highers	127/158	80%	0.048	2.05 (1.01 to 4.17)
	Other higher education	129/170	76%	0.346	1.38 (0.70 to 2.72)
	Degree or equivalent	446/528	84%	0.002	2.68 (1.42 to, 5.07)
Do you have any physical or mental conditions or illnesses lasting or expected to last 12 months or more?					
	No	624/777	80%	–	1
	Yes, but does not reduce activity	82/99	83%	0.704	1.12 (0.63 to 1.98)
	Yes, and reduces activity	198/254	78%	0.528	0.89 (0.61 to 1.29)
Is there anyone who you look after or give special help to, for example, someone who is sick, has a long-term physical or mental disability or is elderly?					
	No	670/829	81%		1
	Yes	195/246	79%	0.431	0.86 (0.58 to 1.26)
	Yes, but only in a professional capacity as part of my job	43/60	72%	0.120	0.61 (0.33 to 1.14)
Do you or does someone close to you have a condition or illness that you think is likely to shorten life?					
	No	647/817	79%		1
	Yes	261/318	82%	0.298	1.22 (0.84 to 1.76)

* Multivariable analysis adjusted for all variables presented.

Half of those people without an ECTP would want one at present (618/1112, 56%) (online supplemental appendix 2, table S4). Age did not have a strong influence on this, although the highest proportion wanting a plan was in the 25–34 years age group (63%) and the lowest in the 65–69 years age group (47%) (online supplemental appendix 2, table S5). People in good health were less likely than those with activity-limiting chronic disease to want a plan at present (52% vs 64%, OR 1.78, 95% CI 1.30 to 2.45, p<0.001) (table 3). Asking a more specific question of when people would want an ECTP, 152/1112 (14%) would want one ‘now’ (table 4). Just 36 (3%) said they would never want one (table 4). Developing a long-term condition or becoming disabled would lead 43% (481/1112) and 42% (467/1112) of individuals, respectively, to want an ECTP. Additionally, 634/1112 (57%) would want a plan if they developed a life-threatening condition (table 4). Of the 40% (441/1127) of people who said they would want an ECTP when were older, the peak decades identified were their 60s and 70s, with 61% (270/441) wanting an ECTP in place by the time they were aged 70 years (online supplemental appendix 2, table S6).

**Table 3:** Would you or would you not like to have an emergency care and treatment plan (ECTP) for yourself at present?* (N=1112)†

		Would like		P value	OR (95% CI)
Gender					
	Male	260/482	54%		
	Female	349/609	57%	0.321	1.14 (0.88 to 1.46)
	Other	2/7	29%	0.180	0.30 (0.05 to 1.73)
Age (years)					
	18–24	39/62	63%	–	1
	25–34	109/168	65%	0.912	1.04 (0.55 to 1.94)
	35–44	90/168	54%	0.164	0.64 (0.35 to 1.20)
	45–54	106/204	52%	0.089	0.59 (0.32 to 1.08)
	55–59	56/98	57%	0.366	0.73 (0.37 to 1.44)
	60–64	61/107	57%	0.275	0.69 (0.35 to 1.35)
	65–69	49/104	47%	0.045	0.50 (0.26 to 0.98)
	70+	108/200	54%	0.195	0.66 (0.35 to 1.24)
Ethnicity					
	White	544/987	55%	–	1
	Black	10/13	77%	0.117	2.91 (0.76 to 11.1)
	Mixed	23/43	53%	0.595	0.84 (0.44 to 1.60)
	Asian	33/54	61%	0.461	1.25 (0.69 to 2.25)
Educational level					
	No qualifications	33/59	56%	–	1
	Qualification less than A level	95/179	53%	0.699	0.89 (0.48 to 1.63)
	A-levels/SCE highers	74/153	48%	0.285	0.71 (0.38 to 1.33)
	Other higher education	93/166	56%	0.810	1.08 (0.58 to 2.00)
	Degree or equivalent	310/521	60%	0.681	1.13 (0.64 to 1.99)
Do you have any physical or mental conditions or illnesses lasting or expected to last 12 months or more?					
	No	400/765	52%	–	1
	Yes, but does not reduce activity	56/97	58%	0.159	1.37 (0.88 to 2.14)
	Yes, and reduces activity	158/245	64%	<0.001	1.78 (1.30 to 2.45)
Is there anyone who you look after or give special help to, for example, someone who is sick, has a long-term physical or mental disability or is elderly?					
	No	461/816	56%		
	Yes	130/238	55%	0.481	0.89 (0.65 to 1.23)
	Yes, but only in a professional capacity as part of my job	27/58	47%	0.054	0.57 (0.32 to 1.01)
Do you or does someone close to you have a condition or illness that you think is likely to shorten life?					
	No	437/805	54%		
	Yes	181/307	59%	0.348	1.15 (0.86 to 1.55)

* Multivariable analysis adjusted for all variables presented.

† Denominator is 1112 people without an ECTP, outcome is sum of definitely would and probably would.

**Table 4:** When, if ever, do you think you would like to have an emergency care and treatment plan in place for yourself? (N=1112)

	Yes, n (%)	No, n (%)	Do not know/refused
Now	152 (14)	940 (85)	20 (2)
Never	36 (3)	1056 (95)	20 (2)
When I am older	441 (40)	651 (59)	20 (2)
If I get diagnosed with a life-threatening condition	634 (57)	458 (41)	20 (2)
If I had a chronic long-term condition	481 (43)	611 (55)	20 (2)
If I were to become severely disabled	467 (42)	625 (56)	20 (2)
Other	12 (1)	1080 (97)	20 (2)

Denominator is 1112 respondents who answered ‘no’ when asked if they had an emergency care and treatment plan.

Most people, 698/1112 (63%), felt they would be at least fairly comfortable making an ECTP; just 49/1112 (4%) felt they would be very uncomfortable making an ECTP (online supplemental appendix 2, table S7). Educational level had a substantial effect on how comfortable respondents thought they would be making an ECTP; 49% in those with no qualifications compared with 70% in those with degrees (p=0.002) (table 5). The results of post hoc sensitivity analyses using backward elimination models were broadly similar to the regression analyses reported here (online supplemental appendix 3). Unadjusted analyses are available in online supplemental appendix 4.

**Table 5:** How comfortable or uncomfortable do you feel about making an emergency care and treatment plan (ECTP) yourself with a doctor or nurse?* (N=1112)†

		Comfortable		P value	OR (95% CI)
Gender					
	Male	309/482	64%	–	1
	Female	380/609	62%	0.647	0.94 (0.72 to 1.22)
	Other	2/7	29%	0.118	0.25 (0.05 to 1.41)
Age (years)					
	18–24	40/62	65%	–	1
	25–34	116/168	69%	0.977	1.01 (0.53 to 1.92)
	35–44	100/168	60%	0.197	0.66 (0.35 to 1.24)
	45–54	117/204	57%	0.141	0.63 (0.34 to 1.17)
	55–59	66/98	67%	0.822	1.08 (0.53 to 2.21)
	60–64	73/107	68%	0.885	1.05 (0.52 to 2.13)
	65–69	65/104	63%	0.487	0.78 (0.39 to 1.56)
	70+	121/200	61%	0.359	0.74 (0.39 to 1.41)
Ethnicity					
	White	633/987	64%	–	1
	Black	6/13	46%	0.092	0.38 (0.12 to 1.17)
	Mixed	22/43	51%	0.097	0.58 (0.30 to 1.11)
	Asian	32/54	59%	0.354	0.76 (0.42 to 1.36)
Educational level					
	No qualifications	29/59	49%	–	1
	Qualification less than A level	91/179	51%	0.792	1.08 (0.59 to 1.99)
	A-levels/SCE highers	94/153	61%	0.064	1.82 (0.97 to 3.44)
	Other higher education	100/166	60%	0.174	1.53 (0.83 to 2.84)
	Degree or equivalent	365/521	70%	0.002	2.48 (1.40 to 4.40)
Do you have any physical or mental conditions or illnesses lasting or expected to last 12 months or more?					
	No	479/765	63%	–	1
	Yes, but does not reduce activity	74/97	59%	0.013	1.91 (1.14 to 3.19)
	Yes, and reduces activity	144/245	59%	0.452	0.89 (0.65 to 1.21)
Is there anyone who you look after or give special help to, for example, someone who is sick, has a long-term physical or mental disability or is elderly?					
	No	512/816	63%		
	Yes	154/238	65%	0.570	1.10 (0.79 to 1.53)
	Yes, but only in a professional capacity as part of my job	32/58	58%	0.310	0.74 (0.42 to 1.32)
Do you or does someone close to you have a condition or illness that you think is likely to shorten life?					
	No	494/805	61%		
	Yes	204/307	66%	0.193	1.23 (0.90 to 1.67)

* Multivariable analysis adjusted for all variables presented.

† Denominator is 1112 people who answered ‘no’ when asked if they had an ECTP outcome is sum of very comfortable and comfortable.

Predominantly, 938/1135 (83%) respondents agreed or agreed strongly that ECTPs would help avoid their family needing to make difficult decisions on their behalf, and that a plan would ensure doctors and nurses knew their wishes, 939/1135 (83%) (table 6). Nevertheless, a small majority, 628/1135 (55%), agreed that there was a serious risk that the plan could be out of date and not reflect the person’s current views or health condition. A substantial minority, 330/1135 (29%), agreed that they might not get life-saving treatment (table 6). Half (316/618, 51%) of those who wanted an ECTP would want their GP to complete it whilst a quarter (161/618, 26%) would want a doctor/nurse who did not know them but who was trained in making ECTPs to complete the plan with them (online supplemental appendix 2, table S3).

**Table 6:** Please say how much you agree or disagree with the following statements about having and emergency care and treatment plan (n=1135)

	*I might not get the treatment that could save my life (n, %)*	*Having a plan can avoid my family having to make difficult decisions for me (n, %)*	*There is a serious risk that the plan could be out of date and not reflect my current views or my current health condition (n, %)*	*Having a plan ensures that doctors and nurses know my wishes (n, %)*
Strongly agree	66 (6)	302 (27)	90 (8)	227 (20)
Agree	264 (23)	636 (56)	538 (47)	712 (63)
Neither agree nor disagree	445 (39)	143 (13)	346 (30)	142 (13)
Disagree	256 (23)	20 (2)	124 (11)	29 (3)
Strongly disagree	89 (8)	22 (2)	23 (2)	13 (1)
Do not know/refused	15 (1)	12 (1)	14 (1)	12 (1)

## Discussion

To our knowledge, this is the first large, representative public survey of the Great British public’s views and attitudes to the completion of ECTPs. The findings show that the Great British public are generally supportive of ECTPs and over 50% would want one for themselves. People with experience of an activity limiting chronic disease are more likely to want one for themselves. The preferred timing for completion of an ECTP was predominantly related to having a life-limiting or life-threatening disease, a chronic illness or being over 60 years. Most respondents would feel at least fairly comfortable making an ECTP and GPs were the preferred healthcare professional to complete a plan with. Most respondents agreed that ECTPs would help to communicate their wishes about future treatments, but over half agreed that the plan might not accurately reflect their wishes at the time of use.

It is striking that that respondents aged 18–34 years are more likely to want an ECTP for themselves at present than those aged over 65 years. Indeed, 63% of respondents aged 18–24 years wanted an ECTP compared with just 47% of respondents aged 65–69 years (online supplemental appendix 2, table S5). Some caution is needed when interpreting these data since when participants were asked when they might want an ECTP in place for themselves, overall, just 14% responded ‘now’ (table 4). The two questions have been interpreted differently. It is not clear why this might be, but it is possible that respondents would like a plan for themselves at some point, but do not feel a need for a plan in their current circumstances. This would be consistent with most respondents identifying the diagnosis of a life-threatening or long-term chronic disease as a trigger for an ECTP completion. Respondents in the 18–24 years age group were also more likely to feel comfortable with an ECTP discussion. These findings related to age are perhaps counterintuitive as younger people in general are less likely to need, or to have considered, an ECTP. Less surprisingly, people with a degree were the most likely group to feel comfortable with an ECTP discussion. Personal experience of a long-term condition that reduced daily activities was associated with being more likely to want to have an ECTP in place. However, experience of knowing or caring for someone with a life-shortening illness was not significantly associated with attitudes to having an ECTP for oneself. Further research to explore the factors that influence attitudes to and comfort with, ECTPs, including age, would be illuminating.

The percentage of respondents who would like to have an ECTP in place for themselves contrasts sharply with studies of public attitudes to advance care planning which predominantly find a reluctance to engage with advance care planning, despite recognising the benefits.^20 24^ A perception that advance care planning (ACP) is primarily associated with end-of-life care is often cited as a reason for this reluctance. It may be that the focus of ECTPs on emergency medical treatment recommendations rather than broader choices related to end-of-life care, removes or reduces this perception.

Respondents were comfortable with a range of healthcare professionals who might, or might not know them, completing the forms. A substantial minority would prioritise a clinician who was knowledgeable about the ReSPECT process over a clinician who knows them personally. In our survey of GP attitudes to ECTPs, we found that many GPs were supportive of a much wider range of health and social care professionals, including care home staff, completing ECTPs.^10^ The literature on ACP shows that nurses are often involved in ACP particularly in secondary care, and programmes to train nursing home staff to support ACP have been reported.^25^,^29^ However, we were unable to identify any studies or reviews that looked at patient or public preference regarding who they would wish to have an ACP conversation with. In expanding the range of health and social care professionals who can facilitate ECTP discussions, it will be important to evaluate the impact of different approaches on the quality of the ECTP discussions, patient experience and resultant plans.

The overall positive attitudes to ECTPs identified in this survey are perhaps surprising, given that over a quarter (29%) of people thought an ECTP might lead to them being denied life-saving treatment, and more than half (55%) thought there was a serious risk it could be out of date when needed (table 6). A 2021 study from Northern Ireland found that 14% of people were worried that having an advance care plan in place might mean treatment was stopped too soon.^20^ From our study, we do not know if this denial of life-saving treatment would be clinically appropriate. These mismatches might be important when ECTPs are being discussed. The perception that there might be a combination of the serious risk of the ECTP being out of date, not reflecting the person’s current views or health condition, and a risk that it may lead to life-saving treatment being denied might substantially reduce their acceptability to the public.

These survey data have allowed us to document public attitudes to ECTPs but does not give any explanation for the findings. Some caution is needed when interpreting statistical significance in our regression models because of the large number of analyses done.

### Strengths and limitations

The use of the annual BSA survey to collect these data means we can be confident that our sample is nationally representative, and that the data quality is good. However, this approach will inevitably exclude many for whom ECTPs are most relevant; that is, frail older people and those with cognitive impairment. As might be expected with a ‘push’ survey of this nature, minority ethnic groups are slightly under-represented. The survey is not, however large enough, or designed in a manner, to allow us to explore if there are any differences in attitudes to emergency care and treatment plans by ethnic group. The finding that people of Asian ethnicity might be slightly less likely to favour everyone having an ECTP may be no more than a chance observation because of the large number of comparisons made. The process for developing our questions, and their piloting by the BSA survey team reduces any ambiguity in the questions. Nevertheless, there remains a possibility that there might have been some misunderstanding of the purpose of each question.

Caution is also needed interpreting the data on completed ECTP forms because of the small numbers, and that they are commonly completed for people with cognitive impairment who might have been less likely to be approached for the survey.

In conclusion, we have documented general support for the use of ECTPs by people of all ages. Nevertheless, some data suggested that people felt these might become seriously out of date, not reflecting the person’s current views or health condition when needed and might prevent people receiving life-saving treatment.

## supplementary material

10.1136/bmjopen-2023-080162 online supplemental file 1

10.1136/bmjopen-2023-080162 online supplemental file 2

10.1136/bmjopen-2023-080162 online supplemental file 3

10.1136/bmjopen-2023-080162 online supplemental file 4

## Data Availability

The data for these analyses are from the NatCEN Social Researcher’s British Social Attitudes Survey, National Centre for Social Research 2023. Archived data are available at ukdataservice.ac.uk. For archive or dataset requests, please email BSA@natcen.ac.uk.
