# Supplementary material for: Public attitudes to emergency care treatment plans: a population survey of Great Britain
Source: BMJ Open. 2024 Sep 23;14(9):e080162. doi: 10.1136/bmjopen-2023-080162 (PMC11429361; doi:10.1136/bmjopen-2023-080162)
Supplement: online supplemental file 1 [file bmjopen-14-9-s001.pdf]

Appendix 1

BSA 2022 Emergency Care and Treatment Plan module

Please not these questions will form part of a larger survey and will be delivered eihter online or by a telephone interviewer depending on the participant's preference. Therefore, the formatting and presentation will be different in practice. And not all participants will be asked all questions. This represents the content of the questions.

Text with explanation of Emergency Care Treatment Plans

The next few questions are about health care and treatment in emergency situations.

Remember, you do not have to answer any of the questions if you do not wish to.

Sometimes when people have an emergency or are very ill, they are so unwell that they can't tell doctors and nurses what care and treatment they want.

An 'Emergency Care and Treatment plan' can be made for anyone, as part of NHS care.

It is a form that is filled out by a doctor or nurse with you, before an emergency happens or you become too unwell. It can be updated as needed.

The form is kept with you and records:

- Your current health.
- What you value most from your treatment and care, such as living as long as possible, keeping your independence, being free from pain.
- What treatments would be recommended for you to have, or to avoid, should you become seriously unwell.

These plans are recommendations and not legally binding.

Q1. Are you in favour or against anyone being able to have an Emergency Care and Treatment Plan if they wish?

1. Strongly in favour
2. Somewhat in favour
3. Neither in favour nor against
4. Somewhat against
5. Strongly against

SPONTANEOUS (Don't know)
SPONTANEOUS (Prefer not to answer)

Q2. Do you currently have an Emergency Care and Treatment Plan in place for **yourself** (that was completed by a doctor or a nurse)?

1. Yes
2. No

SPONTANEOUS (Don't know)
SPONTANEOUS (Prefer not to answer)

If answer is yes then go to Q3; If answer is no then go to Q5

Q3. Who made your Emergency Care and Treatment Plan with you?

1. My GP
2. Another doctor who knows me and my health well
3. A nurse in my GP's surgery
4. Another nurse who knows me and my health well
5. A doctor or nurse who does not know me but is trained in making an Emergency Care and Treatment plan
6. Other – please specify

SPONTANEOUS (Don't know)
SPONTANEOUS (Prefer not to answer)

Q4. "When did you have your Emergency Care and Treatment Plan completed?

1. When I reached a certain age (please specify)
2. After I got diagnosed with a life-threatening condition
3. After I was told I had a chronic long-term condition
4. After I became severely disabled
5. Other, please specify

SPONTANEOUS (Don't know)
SPONTANEOUS (Prefer not to answer)

Q5. Would you or would you not like to have an Emergency Care and Treatment Plan for **yourself** at present?

1. Definitely would
2. Probably would
3. Probably would **not**
4. Definitely would **not**

SPONTANEOUS (Don't know)
SPONTANEOUS (Prefer not to answer)

 This project is funded by the National Institute for Health Research (NIHR) Health Services and Delivery Research programme

Q6. If you were to have an Emergency Care and Treatment Plan completed tomorrow, who would you prefer to discuss it with?

1. My GP
2. A nurse in my GP surgery
3. Another doctor who knows me and my health well
4. Another nurse who knows me and my health well
5. A doctor or nurse who does not know me but is trained in making an Emergency Care and Treatment plan
6. Other – please specify

SPONTANEOUS (Don't know)
SPONTANEOUS (Prefer not to answer)

Q7. When, if ever, do you think you would like to have an Emergency Care and Treatment Plan in place for **yourself**?

1. Now
2. Never
3. When I am older
4. If I get diagnosed with a life-threatening condition
5. If I had a chronic long-term condition
6. If I were to become severely disabled
7. Other, please specify

SPONTANEOUS (Don't know)
SPONTANEOUS (Prefer not to answer)

IF answer to Q7 is 3 ask the following question

Q7. "You said you would you like to have an Emergency Care and Treatment when you are older. At what age?

1. 18-30 years old
2. 31-40 years old
3. 41-50 years old
4. 51-60 years old
5. 61-70 years old
6. 71-80 years old
7. 81-90 years old
8. Over 90 years old

SPONTANEOUS (Don't know)
SPONTANEOUS (Prefer not to answer)

Q8. How comfortable or uncomfortable If Q1 = 1 (Yes)“did you” *if Q1=2 (No)* “would you”} feel about making an Emergency Care and Treatment Plan **yourself** with a doctor or nurse?

1. Very comfortable
2. Fairly comfortable
3. Neither comfortable nor uncomfortable
4. Fairly uncomfortable
5. Very uncomfortable

SPONTANEOUS (Don’t know)
SPONTANEOUS (Prefer not to answer)

Q9. Please say how much you **agree** or **disagree** with the following statements about **having an Emergency Care and Treatment Plan**:

_TEL: “INTERVIEWER: READ OUT EACH STATEMENT AND THE ANSWER CODES. REPEAT ANSWER CODES AS REQUIRED.”

	Strongly agree	Agree	Neither agree or disagree	Disagree	Strongly disagree
I might not get the treatment that could save my life					
Having a plan can avoid my family having to make difficult decisions for me					
There is a serious risk that the plan could be out of date and not reflect my current views or my current health condition					
Having a plan ensures that doctors and nurses know my wishes					

Q10. Have you had an experience where, looking back, you wish there had been an Emergency Care and Treatment Plan in place for a close family member?

1. Yes
2. No

Q11. Would you or would you not like to be involved in having an Emergency Care and Treatment Plan completed for **a close family member**, if they were not able to do so themselves?

1. Definitely would
2. Probably would
3. Probably would **not**
4. Definitely would **not**

SPONTANEOUS (Don't know)
SPONTANEOUS (Prefer not to say)

Q12. Do you have any physical or mental conditions or illnesses lasting or expected to last 12 months or more?

1. Yes
2. No

SPONTANEOUS (Don't know)
SPONTANEOUS (Prefer not to answer)

If answered 1 (yes) to Q12 then ask Q13

Q13. Do any of your conditions or illnesses reduce your ability to carry out day-to-day activities?

1. Yes, a lot
2. Yes, a little
3. Not at all

SPONTANEOUS (Don't know)
SPONTANEOUS (Prefer not to answer)

Q14. Is there anyone who you look after or give special help to, for example, someone who is sick, has a long-term physical or mental disability or is elderly? This may be a spouse, partner, other relative or friend and may be someone living with you or someone who lives elsewhere?

1. Yes
2. Yes, but only in a professional capacity as part of my job
3. No

SPONTANEOUS (Don't know)
SPONTANEOUS (Prefer not to answer)

Q15. Do you or does someone close to you have a condition or illness that you think is likely to shorten life?

1. Yes
2. No

SPONTANEOUS (Don't know)
SPONTANEOUS (Prefer not to answer)
