# Supplementary material for: Public attitudes to emergency care treatment plans: a population survey of Great Britain
Source: BMJ Open. 2024 Sep 23;14(9):e080162. doi: 10.1136/bmjopen-2023-080162 (PMC11429361; doi:10.1136/bmjopen-2023-080162)
Supplement: online supplemental file 2 [file bmjopen-14-9-s002.pdf]

Appendix 2

Supplementary results tables

Table S1

Participant characteristics N=1135

	n	%
Gender		
Male	497	44%
Female	638	56%
Age		
18 to 24	66	6%
25 to 34	173	15%
35 to 44	172	15%
45 to 54	205	18%
55 to 59	99	9%
60 to 64	107	9%
65 to 69	107	9%
70 or over	205	18%
Missing	1	
Location		
South East	166	15%
South West	126	11%
North West	114	10%
East of England	107	9%
Yorkshire and Humber	107	9%
London	106	9%
Scotland	105	9%
West Midlands	100	9%
East Midlands	96	8%
North East	59	5%
Wales	49	4%
Ethnicity		
White Origin	1,005	89%
Asian Origin	55	5%
Mixed Origin	44	4%
Black Origin	14	1%
Don't Know	7	1%
Missing	10	1%
Highest educational level		
No qualifications	61	5%
Below A level[a]	183	16%
A-levels/SCE Highers[b]	158	14%
Other Higher Education[c]	170	15%
Degree	528	47%
Other	21	2%
Missing	14	1%
Have you had an experience where, looking back, you wish there had been an Emergency Care and Treatment Plan in place for a close family member?		
Yes	267	24%
No	859	76%

Don’t know or refused	9	1%
Do you have any physical or mental conditions or illnesses lasting or expected to last 12 months or more?		
No	777	68%
Yes, but does not reduce activity	99	9%
Yes, and reduces activity	254	22%
Don’t know or refused	5	<1%
Is there anyone who you look after or give special help to, for example, someone who is sick, has a long-term physical or mental disability or is elderly?		
No	818	72%
Yes	246	22%
Yes, but only in a professional capacity as part of my job	60	5%
Don’t know / refused	11	1%
Do you or does someone close to you have a condition or illness that you think is likely to shorten life?		
No	808	71
Yes	318	28%
Don’t know or refused	9	1%

a, e.g. GCSE / O level; b, including vocational level 3 or equivalent, and above; c, including Diplomas in Higher Education, HNC and HND; d, or equivalent, and above

Table S2
When did you have your Emergency Care and Treatment Plan completed?
N=17

	Yes, n (%)	No, n (%)	Refused, n(%)
When I reached a certain age	3 (18)	13 (76)	1 (6)
After I got diagnosed with a life-threatening condition	7 (41)	9 (53)	1 (6)
After I was told I had a long-term condition	6 (35)	10 (59)	1 (6)
After I became severely disabled	4 (24)	12 (71)	1 (6)
Other	1 (6)	15 (88)	1 (6)

Table S3
Emergency care and treatment plan completion

	Who made your emergency care treatment plan with you? N=17 (%)	If you were to have an emergency care and treatment plan completed tomorrow, who would you prefer to discuss it with?[a] N= 618 (%)
My GP	6 (35)	316 (51)
Another Doctor who knows me well	4 (24)	59 (10)
A nurse at my GP surgery	2 (12)	46 (7)
A doctor or nurse who does not know me but is trained in making emergency care and treatment plans	2 (12)	161 (26)
Family member	-	18 (3)
Other	3 (18)	5 (1)
Don't know / prefer not to answer	-	6 (1)

a. Denominator is 618 people who would definitely, or probably, want an Emergency Care and treatment plan.

Table S4

Attitudes to Emergency Care and Treatment plan completion

	Would you or would you not like to have an Emergency Care and Treatment Plan for **yourself** at present? N=1,112[a]	Would you or would you not like to be involved in having an Emergency Care and Treatment Plan completed for **a close family member**, if they were not able to do so themselves? N=1,135
Definitely would	128 (12)	302 (27)
Probably would	490 (44)	628 (55)
Probably would not	369 (33)	138 (12)
Definitely would not	102 (9)	41 (4)
Don't know / refused	23 (2)	26 (2)

a. Denominator is 1,112 respondents who answered 'no' when asked if they had an emergency care and treatment plan

Table S5

**Would you or would you not like to have an Emergency Care and Treatment Plan for yourself at present?**?			
18-24	39	62	63%
25-34	99	168	59%
35-44	90	168	54%
45-54	106	204	52%
55-69	56	98	57%
60-64	61	107	57%
65-69	49	104	47%
>70	108	200	54%
All	608	1111	55%

Denominator is 1111 people without a current emergency care and treatment plan who gave a valid response

Table S6

You said you would like to have an Emergency Care and treatment (plan) when you are older. At what age?

N=441

	n (%)	Cumulative %
18 - 30	4 (10)	1
31 - 40	17 (4)	5
41 - 50	39 (9)	14
51 - 60	65 (15)	28
61 - 70	145 (33)	61
71 - 80	119 (27)	88
81 - 90	40 (9)	97
Over 90	0 (0)	97
Don’t know / refused	12 (3)	100

Denominator is 414 respondents who indicated they would want an emergency care and treatment plan when they got older

Table S7
How comfortable or uncomfortable do you feel about making an Emergency Care and Treatment Plan yourself with a doctor or nurse? (N=1,112)[a]

Very comfortable	260 (23)
Fairly comfortable	438 (39)
Neither comfortable nor uncomfortable	233 (21)
Fairly uncomfortable	123 (11)
Very uncomfortable	49 (4)
Don’t know / refused	9 (1)

a. Denominator is 1,112 respondents who answered ‘no’ when asked if they had an emergency care and treatment plan.
