# Supplementary material for: Public attitudes to emergency care treatment plans: a population survey of Great Britain
Source: BMJ Open. 2024 Sep 23;14(9):e080162. doi: 10.1136/bmjopen-2023-080162 (PMC11429361; doi:10.1136/bmjopen-2023-080162)
Supplement: online supplemental file 3 [file bmjopen-14-9-s003.pdf]

**Appendix 3**

**Backward Elimination Analyses**

**Table 1**
**Are you in favour or against anyone being able to have an Emergency Care and Treatment Plan if they wish?**[a]
*N=1135*

| | | *In Favour* | | p-value | OR (95%CI) |
|---|---|---|---|---|---|
| *Ethnicity* | | | | | |
| | White | 820/1005 | 82% | - | 1 |
| | Black | 10/14 | 71% | 0.181 | 0.45(0.14,1.46) |
| | Mixed | 30/44 | 68% | 0.030* | 0.48(0.25,0.93) |
| | Asian | 37/55 | 67% | 0.002* | 0.40(0.22,0.72) |
| *Educational Level* | | | | | |
| | No qualifications | 43/61 | 70% | - | 1 |
| | Qualifications less than A level | 138/183 | 75% | 0.441 | 1.29(0.67,2.47) |
| | A-levels/SCE Highers | 127/158 | 80% | 0.087 | 1.82(0.92,3.6) |
| | Other Higher Education | 129/170 | 76% | 0.417 | 1.31(0.68,2.53) |
| | Degree or equivalent | 446/528 | 84% | 0.004* | 2.44(1.33,4.47) |

a. Backward elimination analysis

**Are you in favour or against anyone being able to have an Emergency Care and Treatment Plan if they wish?**

After performing backward elimination, Asian ethnicity (p=0.002) and degree or equivalent educational level (p=0.004) are the significant variables.

| Table 2<br>**Would you or not like to have an Emergency Care and Treatment Plan for yourself at present?[a]**<br>*N=1,112[b]* | | | | |
|---|---|---|---|---|
| | *Would Like* | | p-value | OR (95%CI) |
| *Educational Level* | | | | |
| No qualifications | 33/59 | 56% | - | 1 |
| Qualification less than A level | 95/179 | 53% | 0.721 | 0.90(0.49,1.63) |
| A-levels/SCE Highers | 74/153 | 48% | 0.360 | 0.75(0.41,1.38) |
| Other Higher Education | 93/166 | 56% | 0.762 | 1.10(0.6,2.01) |
| Degree or equivalent | 310/521 | 60% | 0.426 | 1.25(0.72,2.16) |
| Other | 13/34 | 38% | 0.073 | 0.44(0.18,1.08) |
| *Do you have any physical or mental conditions or illnesses lasting/expected to last 12 months or more?* | | | | |
| No | 400/765 | 52% | - | 1 |
| Yes, but does not reduce activity | 56/97 | 58% | 0.317 | 1.25(0.81,1.92) |
| Yes, and reduces activity | 158/245 | 64% | 0.000* | 1.78(1.31,2.41) |

a. Backward elimination analysis; b, denominator is 1,112 people without an ECTP, outcome is sum of definitely would and probably would

**Would you or not like to have an Emergency Care and Treatment Plan for yourself at present?**

After performing backward elimination, other educational level (p=0.073) and having a long-term illness that reduces activity (p<0.001) are the significant variables.

**Table 3**
**How comfortable or uncomfortable do you feel about making an Emergency Care and Treatment Plan yourself with a doctor or nurse?a**
*N=1,112b*

| | | *Comfortable* | | p-value | OR (95%CI) |
|---|---|---|---|---|---|
| *Ethnicity* | | | | | |
| | White | 633/987 | 64% | - | 1 |
| | Black | 6/13 | 46% | 0.090 | 0.38(0.13,1.16) |
| | Mixed | 22/43 | 51% | 0.111 | 0.6(0.32,1.12) |
| | Asian | 32/54 | 59% | 0.249 | 0.71(0.4,1.26) |
| | Refused | 5/15 | 33% | 0.029* | 0.29(0.09,0.88) |
| *Educational Level* | | | | | |
| | No qualifications | 29/59 | 49% | - | 1 |
| | Qualification less than A level | 91/179 | 51% | 0.767 | 1.09(0.6,1.98) |
| | A-levels/SCE Highers | 94/153 | 61% | 0.070 | 1.76(0.96,3.25) |
| | Other Higher Education | 100/166 | 60% | 0.161 | 1.54(0.84,2.82) |
| | Degree or equivalent | 365/521 | 70% | 0.001* | 2.48(1.43,4.3) |
| *Do you have any physical or mental conditions or illnesses lasting/expected to last 12 months or more?* | | | | | |
| | No | 479/765 | 63% | - | 1 |
| | Yes, but does not reduce activity | 74/97 | 59% | 0.015* | 1.86(1.13,3.07) |
| | Yes, and reduces activity | 144/245 | 59% | 0.666 | 0.94(0.69,1.27) |

a. Backward elimination analysis; b, denominator is 1,112 people who answered 'no' when asked if they had an ECTP

**How comfortable or uncomfortable do you feel about making an Emergency Care and Treatment Plan yourself with a doctor or nurse?**

After performing backward elimination, refusing to say ethnicity (p=0.029), degree or equivalent educational level (p=0.001) and having a long-term illness that does not reduce activity (p=0.015) were the remaining significant variables in the model.
