# Supplementary material for: Public attitudes to emergency care treatment plans: a population survey of Great Britain
Source: BMJ Open. 2024 Sep 23;14(9):e080162. doi: 10.1136/bmjopen-2023-080162 (PMC11429361; doi:10.1136/bmjopen-2023-080162)
Supplement: online supplemental file 4 [file bmjopen-14-9-s004.pdf]

Appendix 4
Unadjusted Regression Analyses

Table 1
Are you in favour or against anyone being able to have an Emergency Care and Treatment Plan if they wish?[a] *N=1135*

Gender		*In favour*		p-value	OR (95%CI)
	Male	380/493	77%		1
	Female	510/619	82%	0.028*	1.39(1.04,1.87)
	Other	7/8	88%	0.495	2.08(0.25,17.1)
Age					
	18-24	51/66	77%	-	1
	25-34	137/173	79%	0.746	1.12(0.57,2.22)
	35-44	138/172	80%	0.613	1.19(0.6,2.37)
	45-54	160/205	78%	0.895	1.05(0.54,2.03)
	55-59	85/99	86%	0.159	1.79(0.8,4)
	60-64	86/107	80%	0.626	1.2(0.57,2.54)
	65-69	82/107	77%	0.923	0.96(0.47,2)
	70+	168/205	82%	0.402	1.34(0.68,2.63)
Ethnicity					
	White	820/1005	82%	-	1
	Black	10/14	71%	0.338	0.56(0.17,1.82)
	Mixed	30/44	68%	0.029*	0.48(0.25,0.93)
	Asian	37/55	67%	0.010*	0.46(0.26,0.83)
Qualifications					
	No qualifications	43/61	70%	-	1
	Qualifications less than A level	138/183	75%	0.448	1.28(0.67,2.45)
	A-levels/SCE Highers	127/158	80%	0.118	1.71(0.87,3.37)
	Other Higher Education	129/170	76%	0.408	1.32(0.69,2.53)
	Degree or equivalent	446/528	84%	0.007*	2.28(1.25,4.14)
Do you have any physical or mental conditions or illnesses lasting or expected to last 12 months or more?					
	No	624/777	80%	-	1
	Yes, but does not reduce activity	82/99	83%	0.551	1.18(0.68,2.05)
	Yes, and reduces activity	198/254	78%	0.418	0.87(0.61,1.22)
Is there anyone who you look after or give special help to, for example, someone who is sick, has a long-term physical or mental disability or is elderly?					
	No	670/829	81%	-	1
	Yes	195/246	79%	0.590	0.91(0.64,1.29)
	Yes, but only in a professional capacity as part of my job	43/60	72%	0.089	0.6(0.33,1.08)
Do you or does someone close to you have a condition or illness that you think is likely to shorten life?					
	No	647/817	79%	-	1
	Yes	261/318	82%	0.276	1.2(0.86,1.68)

a. non-adjusted multi-variable analysis

Table 2

Would you or not like to have an Emergency Care and Treatment Plan for yourself at present?[a]

N=1,112[b]

Gender		*Would like*		p-value	OR (95%CI)
	Male	260/482	54%		
	Female	349/609	57%	0.266	1.15(0.9,1.46)
	Other	2/7	29%	0.202	0.34(0.07,1.78)
Age					
	18-24	39/62	63%	-	1
	25-34	109/168	65%	0.781	1.09(0.6,1.99)
	35-44	90/168	54%	0.207	0.68(0.37,1.24)
	45-54	106/204	52%	0.131	0.64(0.36,1.14)
	55-59	56/98	57%	0.470	0.79(0.41,1.51)
	60-64	61/107	57%	0.453	0.78(0.41,1.49)
	65-69	49/104	47%	0.050*	0.53(0.28,1.00)
	70+	108/200	54%	0.218	0.69(0.39,1.24)
Ethnicity					
	White	544/987	55%	-	1
	Black	10/13	77%	0.131	2.71(0.74,9.92)
	Mixed	23/43	53%	0.834	0.94(0.51,1.73)
	Asian	33/54	61%	0.389	1.28(0.73,2.24)
Educational Level					
	No qualifications	33/59	56%	-	1
	Qualification less than A level	95/179	53%	0.702	0.89(0.49,1.61)
	A-levels/SCE Highers	74/153	48%	0.324	0.74(0.40,1.35)
	Other Higher Education	93/166	56%	0.990	1.00(0.55,1.83)
	Degree or equivalent	310/521	60%	0.597	1.16(0.67,1.99)
Do you have any physical or mental conditions or illnesses lasting/expected to last 12 months or more?					
	No	400/765	52%	-	1
	Yes, but does not reduce activity	56/97	58%	0.312	1.25(0.81,1.91)
	Yes, and reduces activity	158/245	64%	0.001*	1.66(1.23,2.23)
Is there anyone who you look after or give special help to, for example, someone who is sick, has a long-term physical or mental disability or is elderly?					
	No	461/816	56%		
	Yes	130/238	55%	0.608	0.93(0.69,1.24)
	Yes, but only in a professional capacity as part of my job	27/58	47%	0.143	0.67 (0.39, 1.14)
Do you or does someone close to you have a condition or illness that you think is likely to shorten life?					
	No	437/805	54%		
	Yes	181/307	59%	0.161	1.21 (0.93, 1.58)

a. non-adjusted multi-variable analysis; b, denominator is 1,112 people without an ECTP, outcome is sum of definitely would and probably would

Table 3
How comfortable or uncomfortable do you feel about making an Emergency Care and Treatment Plan yourself with a doctor or nurse?a
N=1,112b

Gender		*Comfortable*		p-value	OR (95%CI)
	Male	309/482	64%	-	1
	Female	380/609	62%	0.561	0.93(0.72,1.19)
	Other	2/7	29%	0.076	0.22(0.04,1.17)
Age					
	18-24	40/62	65%	-	1
	25-34	116/168	69%	0.514	1.23(0.66,2.27)
	35-44	100/168	60%	0.492	0.81(0.44,1.48)
	45-54	117/204	57%	0.316	0.74(0.41,1.33)
	55-59	66/98	67%	0.712	1.13(0.58,2.22)
	60-64	73/107	68%	0.622	1.18(0.61,2.29)
	65-69	65/104	63%	0.794	0.92(0.48,1.76)
	70+	121/200	61%	0.57	0.84(0.47,1.52)
Ethnicity					
	White	633/987	64%	-	1
	Black	6/13	46%	0.189	0.48(0.16,1.44)
	Mixed	22/43	51%	0.087	0.59(0.32,1.08)
	Asian	32/54	59%	0.468	0.81(0.47,1.42)
		Educational Level			
	No qualifications	29/59	49%	-	1
	Qualification less than A level	91/179	51%	0.822	1.07(0.59,1.93)
	A-levels/SCE Highers	94/153	61%	0.106	1.65(0.9,3.02)
	Other Higher Education	100/166	60%	0.14	1.57(0.86,2.85)
	Degree or equivalent	365/521	70%	0.001*	2.42(1.41,4.17)
Do you have any physical or mental conditions or illnesses lasting/expected to last 12 months or more?					
	No	479/765	63%	-	1
	Yes, but does not reduce activity	74/97	59%	0.009*	1.92 (1.18, 3.14)
	Yes, and reduces activity	144/245	59%	0.282	0.85 (0.63, 1.14)
Is there anyone who you look after or give special help to, for example, someone who is sick, has a long-term physical or mental disability or is elderly?					
	No	512/816	63%		
	Yes	154/238	65%	0.581	1.09(0.81,1.47)
	Yes, but only in a professional capacity as part of my job	32/58	58%	0.252	0.73 (0.43, 1.25)
Do you or does someone close to you have a condition or illness that you think is likely to shorten life?					
	No	494/805	61%		
	Yes	204/307	66%	0.117	1.25 (0.95, 1.64)

a. non-adjusted multi-variable analysis; b, denominator is 1,112 people who answered ‘no’ when asked if they had an ECTP
