# Supplementary material for: Public attitudes to emergency care treatment plans: a population survey of Great Britain
Source: BMJ Open. 2024 Sep 23;14(9):e080162. doi: 10.1136/bmjopen-2023-080162 (PMC11429361; doi:10.1136/bmjopen-2023-080162)
Supplement: Supplementary file 5 [file bmjopen-2023-080162.reviewer_comments.pdf]

PEER REVIEW HISTORY

BMJ Open publishes all reviews undertaken for accepted manuscripts. Reviewers are asked to complete a checklist review form and are provided with free text boxes to elaborate on their assessment. These free text comments are reproduced below.

ARTICLE DETAILS

**TITLE (PROVISIONAL)**	Public attitudes to emergency care treatment plans: a population survey of Great Britain
**AUTHORS**	Underwood, Martin; Noufaily, Angela; Bain, Chris; Harlock, Jenny; Griffiths, Frances; Huxley, Caroline; Perkins, Gavin; Rees, Sophie; Slowther, Anne-Marie

VERSION 1 - REVIEW

**REVIEWER NAME**	Schmiedhofer, Martina
**REVIEWER AFFILIATION**	Charité Universitätsmedizin Berlin, Emergency and Acute Medicine
**REVIEWER CONFLICT OF INTEREST**	I have no competing interests
**DATE REVIEW RETURNED**	24-Feb-2024

**GENERAL COMMENTS**	Thank you very much for the request to review this paper. It deals with a current issue concerning the handling of medical treatment in end-of-life situations with regard to the patient's wishes and the willingness and timing to deal with them. The research question, survey and evaluations are conclusively presented.<br>The results can be integrated into national health care policies by providing targeted information about the meaning and implications of an emergency care treatment plan by GPs or other healthcare institutions.

**REVIEWER NAME**	Ingielewicz, Anna
**REVIEWER AFFILIATION**	Medical University of Gdansk
**REVIEWER CONFLICT OF INTEREST**	None
**DATE REVIEW RETURNED**	14-May-2024

**GENERAL COMMENTS**	It was with great pleasure and satisfaction that I read the study on the survey of public attitudes towards the implementation of emergency care and the treatment plan. The topic is important and worth mentioning to the public, this study is a good prelude to a wider study of this issue.<br>My comments relate to the limitations of the study: the imbalance in terms of ethnic groups and the small number of respondents with a complete ECTP. In addition, this study lacks an answer to the question why young and healthy people, despite wanting ECTP for themselves, do not want it ‘now’.

VERSION 1 – AUTHOR RESPONSE

Reviewer comment	
My comments relate to the limitations of the study: the imbalance in terms of ethnic groups and the small number of respondents with a complete ECTP. In addition, this study lacks an answer to the question why young and healthy people, despite wanting ECTP for themselves, do not want it 'now'	1. We agree that the slight under-representation of minority ethnic groups is a potential limitation. This is acknowledged in the paper. The challenges of obtaining adequate representation from minority ethnic groups in health research is well documented. That the British Social Attitudes survey (the gold standard of population survey series in Britain) was not fully able to address this shows just how difficult this can be. We do not feel that any further comment is needed on this point<br>2. As we describe in our introduction, ECTPs are a fairly recent innovation in the UK with the most commonly used form (ReSPECT) only introduced in 2016.The aim of this study was to find out overall public attitudes to ECTPs in light of this initiative. It is therefore to be expected that only a very small number of respondents will have an ECTP. We have noted the small number in the limitations section of the paper. We do not think any further comment is needed on this point<br>3. Like the reviewer we were surprised by the mismatch between younger respondents wanting an ECTP but not now. We have given our thoughts on possible explanations for why this might be in the discussion. The survey methodology used here does not allow us to explain this finding. We do not think any further comment is needed on this point
Additional changes	1. We have changed one citation from 'submitted for publication' now that this paper has been published. This is new citation 23.<br>2. We have changed our data sharing statement to clarify that the National Centre for Social Research own the copyright for the source data.<br>3. We have added an additional conflict of interest for Professor Slowther that has arisen since this paper was submitted for publication.
